# Mild Clinical Course of Severe Fever with Thrombocytopenia Syndrome Virus Infection in an Elderly Japanese Patient

**DOI:** 10.1155/2014/918135

**Published:** 2014-12-04

**Authors:** Yuko Ohagi, Shinobu Tamura, Chiaki Nakamoto, Hiromichi Nakamoto, Masayuki Saijo, Masayuki Shimojima, Yoshio Nakano, Tokuzo Fujimoto

**Affiliations:** ^1^Department of Internal Medicine, Kinan Hospital, 46-70 Shinjo, Tanabe-shi, Wakayama 646-8588, Japan; ^2^Department of Hematology/Oncology, Kinan Hospital, 46-70 Shinjo, Tanabe-shi, Wakayama 646-8588, Japan; ^3^Department of Nursing, Kinan Hospital, 46-70 Shinjo, Tanabe-shi, Wakayama 646-8588, Japan; ^4^Department of Central Clinical Laboratory, Kinan Hospital, 46-70 Shinjo, Tanabe-shi, Wakayama 646-8588, Japan; ^5^Special Pathogens Laboratory, Department of Virology 1, National Institute of Infectious Diseases, 4-7-1 Gakuen, Musashimurayama, Tokyo 208-0011, Japan

## Abstract

Severe fever with thrombocytopenia syndrome (SFTS) is an emerging infectious and hemorrhagic disease recently described in China and western Japan. A 71-year-old healthy Japanese woman noticed a tick biting her after harvesting in an orchard and removed it herself. She developed diarrhea, anorexia, and chills eight days later. Because these symptoms continued, she visited a primary care physician 6 days after the onset. Laboratory data revealed thrombocytopenia, leukocytopenia, and elevated liver enzymes. She was then referred to our hospital. Although not completely fulfilling the diagnostic criteria used in a retrospective study in Japan, SFTS was suspected, and we detected SFTS virus in the patient's blood using RT-PCR. However, she recovered without intensive treatment and severe complications 13 days after the onset. In this report, we present a mild clinical course of SFTS virus infection in Japan in detail.

## 1. Introduction

An outbreak of an unknown infectious disease characterized by high fever, gastrointestinal symptoms, thrombocytopenia, and leukocytopenia occurred between March and July 2009 in China, with a high fatality rate (30%). The emerging infectious disease was named severe fever with thrombocytopenia syndrome (SFTS) based on its clinical characteristics. In 2011, Yu et al. identified a tick-borne infectious disease caused by the SFTS virus (SFTSV), a* Phlebovirus* from the Bunyaviridae family [1]. In Japan, a female patient who lived in the Yamaguchi prefecture was the first to be diagnosed with SFTS in Japan in 2012, and she eventually died due to multiple organ failure (MOF) [2]. The study was retrospectively performed in Japan between 2005 and 2012 and included ten additional patients newly diagnosed with SFTS. All SFTS patients were aged over 50 years and lived in western Japan. Six of these patients eventually died, resulting in a higher fatality rate than that in China [2]. Until June 2014, 67 patients in Japan had been diagnosed with SFTS, and 22 had fatal outcomes. This emerging infectious disease has been gradually introduced to the Japanese population. A recent Infectious Agents Surveillance Report published by the National Institute of Infectious Disease (NIID) stated that a SFTS case not meeting all diagnostic criteria would have a good outcome [3]. Here we report a case of an elderly Japanese patient with a mild clinical course of SFTS in the easternmost region of Japan.

## 2. Case Presentation

In early June, a 71-year-old previously healthy woman, who engaged in agricultural activities in a hilly rural area, noticed a tick biting her left thigh while bathing after harvesting in an orchard. The species of the tick remains unclear. She properly removed the head of the tick herself. However, she suddenly developed watery diarrhea (4-5 times a day), anorexia, and left inguinal lymphadenopathy eight days later. She also felt cold but did not measure her body temperature. Because these symptoms did not improve, particularly her gastrointestinal symptoms, she visited a primary care physician 4 days after the onset of the illness. Laboratory data revealed low platelet and white blood cell counts as well as elevated liver enzymes (Table 1). Although the watery diarrhea and chills gradually improved, she was admitted to our hospital seven days after the onset of the illness due to anorexia.

**Figure 1:**
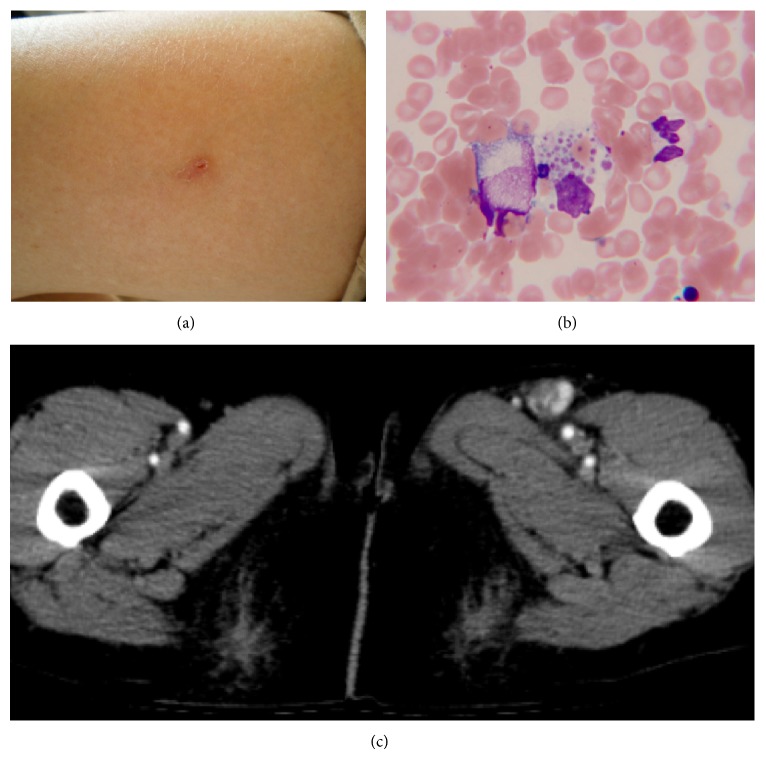
Clinical signs of an elderly patient with SFTSV infection. (a) A tick bite was found on the left thigh. (b) Microscopic findings of Giemsa staining of bone marrow showed platelet-specific hemophagocytes. (c) Contrast-enhanced computed tomography showed left inguinal lymphadenopathy alone.

**Figure 2:**
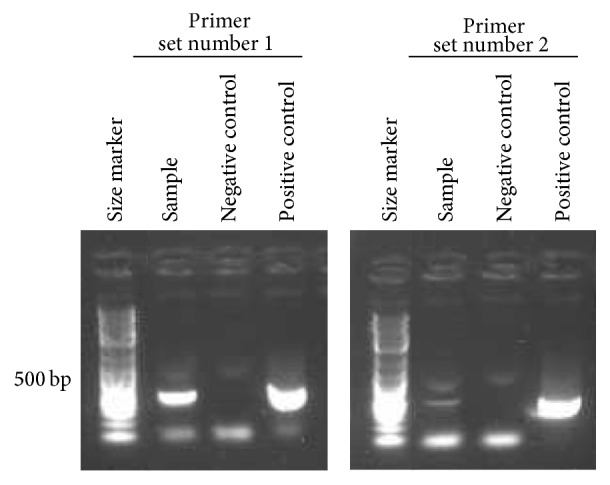
Detection of Japanese SFTSV mRNA using a conventional one-step RT-PCR method. Clinical specimen by RT-PCR: lane 1: our patient's blood sample; lane 2: negative control (NTC); lane 3: positive control (PC) (SFTSV strain HB29 viral RNA). Primer sets numbers 1 and 2 amplified the gene coding SFTSV NP, and the sizes of these products were 458 bp and 461 bp, respectively.

On admission, the patient had a slight headache, but no fever or neurological symptoms. A left inguinal lymph node was swollen, but indolent, whereas none of the other superficial lymph nodes or tonsils were enlarged. The patient did not exhibit erythema, wheal, or petechial rash. A small tick bite was observed in the lateral aspect of her left thigh (Figure 1(a)). Laboratory tests on admission also showed a low platelet count of 7.3 × 10^4^/*μ*L. Coagulation data did not fulfill the diagnostic criteria of disseminated intravascular coagulation (DIC) [4]. White blood cell count was 4.9 × 10^3^/*μ*L, which was within the normal range; however, 15% were atypical lymphocytes. Serum levels of alanine aminotransferase (ALT; 267 IU/L), aspartate aminotransferase (AST; 122 IU/L), and lactate dehydrogenase (LDH; 577 IU/L) were high, whereas creatine kinase (CK), alkaline phosphatase, *γ*-glutamyl transpeptidase, and C-reactive protein (CRP) levels were nearly within normal ranges (Tables 1 and 2). Bone marrow aspiration revealed a mild hypocellular marrow with platelet-specific hemophagocytosis (Figure 1(b)). Contrast-enhanced computed tomography showed significant left inguinal lymphadenopathy (Figure 1(c)). After a few days, serological results confirmed that this disease was not an acute infection by Epstein-Barr virus, cytomegalovirus, or scrub typhus. Although her clinical course did not fulfill the full diagnostic criteria for SFTS, which were previously used in a retrospective study [2], we suspected SFTS and sent her peripheral blood on admission to NIID. The patient had mild hypovolemia in the absence of electrolyte imbalance, and replacement therapy with an adequate infusion fluid was initiated. Minocycline (200 mg/day) was administered intravenously because* Rickettsia tsutsugamushi* was also included in the differential diagnosis. On the 5th day of her admission, serum IgM antibodies to SFTSV were detected by enzyme-linked immunosorbent assay (ELISA), and the SFTSV nucleoprotein (NP) gene was detected by conventional one-step reverse-transcription polymerase chain reaction (RT-PCR; Figure 2). In addition, the level of the SFTSV genome in her blood was 10^4.86^ copies/mL, as determined using quantitative RT-PCR. These results supported the definitive diagnosis of SFTSV infection. We submitted a notification of the occurrence of this infectious disease to the public health center. The administration of minocycline was discontinued after the definitive diagnosis. Platelet count became markedly elevated 10 days after the onset of the illness (Table 1). Serum transaminases returned to almost normal levels 13 days after the onset (Table 1). Her anorexia also simultaneously recovered, and she was subsequently discharged. She never developed fever during her hospitalization.

## 3. Discussion

In a retrospective study performed in Japan, SFTS was defined as a case in which all of the following seven requirements were met: (1) fever of 38°C or higher, (2) digestive symptoms, (3) thrombocytopenia, (4) leukocytopenia, (5) elevated AST/ALT/LDH levels, (6) no other clear cause, and (7) intensive care required or death [2]. The degree of fever in the present case remained unclear before her admission, and she did not require intensive care throughout the clinical course. Although this did not reflect typical SFTS, clinical characteristics such as hemophagocytosis and unilateral lymphadenopathy were consistent with SFTS [1, 2, 5, 6]. Therefore, we performed tests to identify SFTSV. A definitive diagnosis was ultimately reached on the 5th day after admission (12th day from the onset), when her serum samples were found to be positive for IgM antibodies to SFTSV, and the SFTSV NP gene was detected.

In the initial medical examination, it was difficult to distinguish the present case from a* Rickettsia* infection, which is relatively common in this area; therefore, we continued to administer tetracycline antibiotics until a definitive diagnosis could be reached. Because no curative treatment is currently present for SFTS, symptomatic and supportive therapies are typically performed, with systemic antibiotics being used to treat secondary infections [5]. In this present case, we accordingly administered symptomatic and supportive treatments after reaching a definitive diagnosis of SFTS. Throughout the entire course, the patient did not require intensive care, and her symptoms improved with fluid replacement for dehydration alone, suggesting that she had a mild clinical course of SFTS. In China, previous studies have shown that administration of ribavirin, an antiviral drug, against a broad range of RNA viruses, could successfully treat some SFTS patients; however, this treatment is currently not considered to be effective [5–8].

The clinical course of SFTS has largely been divided into 4 stages: (1) incubation, (2) fever stage, (3) MOF stage, and (4) convalescence [5]. After the tick bite, the incubation period lasts for 5 to 14 days. The disease then presents with flu-like symptoms such as fever, headache, malaise, muscular pain, and diarrhea [1, 2, 5, 6]. Most patients generally shift into the MOF stage after a fever stage of 5 to 11 days. Physicians are required to exercise extreme caution here because the most severe leukocytopenia and thrombocytopenia have been reported in the MOF stage; both MOF and DIC have been common, causing several fatalities [1, 2, 5, 6]. The present patient was hospitalized 7 days after the onset of the illness and exhibited no fever. These results suggested that she had passed the fever stage and may already have been in the MOF stage. Prognostic factors for SFTS include abnormal levels of AST, LDH, CK, and CK-MB; abnormal neurological findings; bleeding; DIC; and MOF [6]. Our patient did not exhibit any of these factors, thereby supporting a better outcome on admission.

The viral load of SFTSV in peripheral blood decreases slightly in survivors with the infection, whereas a high titer level of 10^8^ copies/mL or more leads to fatal outcomes [5, 9]. Previous studies reported that the mortality risk was higher for cases with a viral load of 10^5^ copies/mL or more in peripheral blood after symptom onset [5, 9]. The viral load in the present case was not periodically measured over time, and it was impossible to accurately compare the viral load reported in China with that of this case because of sequence mismatches between the Chinese and Japanese SFTSV lineages [9]. In Japan, Yoshikawa et al. developed a conventional and quantitative one-step RT-PCR capable of detecting Japanese SFTSV and recently reported that viral loads of less than 10^5^ copies/mL, as found in the present study, were lower than the mean level measured in the survivor group, which is consistent with previous findings reported by Zhang et al. [9, 10]. Thus, the relatively low viral SFTV load in the blood sample of our patient on admission also contributed to her mild clinical course.

As of June 2014, SFTS has been confirmed in areas to the west of our hospital (particularly in the Kyushu and Shikoku regions) [2, 11]. Ticks carrying SFTSV and animals positive for SFTSV antibodies have been detected in various areas in the absence of human SFTS cases [11]. In the Wakayama prefecture, where our hospital is located, the present case is the first report of SFTSV infection developing in the easternmost area of Japan when the disease was diagnosed. Outbreaks of SFTS in China from 2009 onwards have been most common between May and August [1, 5, 12–14]. On the other hand, outbreaks in the Kyushu region were found to be relatively common in the fall and winter in addition to the period between May and August [2, 11]. These findings suggest that future SFTS outbreaks may occur in more eastern regions throughout the entire year. Physicians in Japan should also pay special attention to the diagnosis of SFTS.

Several cases of severe SFTS were reported between 2011 and 2012 in the Xinyang region in China [12]. Most cases were of elderly farmers in the southern and western areas of this region. Furthermore, high-risk areas of SFTSV infection include environments such as shrubs, forests, and rain-fed croplands [12]. SFTS in Japan appears to be characterized by a spread from western to eastern Japan. A detailed study of high-risk areas for SFTS, similar to that in the Xinyang region in China, should be conducted in Japan. Our patient was also an elderly farmer working in an orchard, which represents an environment where SFTS has commonly occurred in China. Thus, orchards appear to be high-risk areas for SFTSV infection and are now being monitored for this infectious disease. Person-to-person transmission by bloodstream infections, which was reported in China, was not observed in the present case [15, 16]. Standard precautions were strictly followed during the patient's hospitalization.

Here we described a patient with a mild clinical course of SFTSV infection in the easternmost area of Japan. Mild cases may not be diagnosed in routine clinical practice and thus may be overlooked. Therefore, the incidence of SFTSV infection may be higher than reported. Early diagnosis, effective treatment, and prevention need to be established to prevent future endemic spread of SFTSV infection.

## Figures and Tables

**Table 1:** Clinical course of hemoglobin, leukocyte and platelet counts, AST, ALT, creatine kinase, LDH, aPTT, prothrombin time, and C-reactive protein after the onset of SFTS.

Laboratory test (reference range)	Day 4	Day 7	Day 8	Day 9	Day 10	Day 13
Hemoglobin, g/dL (11.5–15.3)	14.4	16.4	13.2	12.3	13.1	12.7
Leukocyte, ×10^3^/*μ*L (4–9.5)	3.0	4.9	3.9	4.2	4.2	4.4
Neutrophil, % (39–73)	NA	45	32	61	56	59
Lymphocyte, % (19–50)	NA	36	52	29	35	27
Atypical lymphocyte, % (0)	NA	15	3	0	0	0
Erythroblast, cell/100 WBC (0)	NA	1	0	0	0	0
Platelet, ×10^3^/*μ*L (150–400)	61	73	79	89	241	431
AST, IU/L (11–35)	157	267	192	147	86	42
ALT, IU/L (5–35)	66	122	97	89	82	56
Creatine kinase, IU/L (45–235)	155	207	127	86	52	31
LDH, IU/L (120–230)	483	577	437	356	284	206
aPTT, sec (20–35)	NA	33.8	31.3	30.5	29.3	26.9
Prothrombin time, INR (0.8–1.2)	NA	0.88	0.89	0.92	0.98	1.03
C-reactive protein, mg/dL (0–0.5)	1.10	0.23	0.15	0.26	0.29	0.04

^*^Day 1 was onset day. NA: not available; WBC: white blood cell; AST: aspartate aminotransferase; ALT: alanine aminotransferase; LDH: lactate dehydrogenase; aPTT: activated partial prothromboplastin time; INR: international normalized ratio.

**Table 2:** Laboratory data of a Japanese elderly patient with mild SFTS on the admission.

*Complete blood count *	
White blood cell	4,900/*μ*L
Neutrophil	45.0%
Lymphocyte	36.0%
Monocyte	4.0%
Eosinophil	0.0%
Basophil	0.0%
Atypical lym.	15.0%
Erythroblast	1 cell/100 WBC
Red blood cell	561 × 10^4^/*μ*L
Hemoglobin	16.4 g/dL
Hematocrit	47.7%
Platelet	7.3 × 10^4^/*μ*L
*Coagulation system *	
aPTT	33.8 sec
PT (%)	166%
PT-INR	0.88
Fibrinogen	246 mg/dL
FDP	4.34 *μ*g/mL
*Chemistry *	
Creatinine	0.90 mg/dL
BUN	22.0 mg/dL
Sodium	136 mEq/L
Potassium	3.9 mEq/L
Chloride	97 mEq/L
AST	267 IU/L
ALT	122 IU/L
ALP	202 IU/L
*γ*-GTP	52 IU/L
T-Bil	0.5 mg/dL
LDH	577 IU/L
Creatine Kinase	207 IU/L
Total Protein	6.2 g/dL
Albumin	3.5 g/dL
CRP	0.23 mg/dL
Glucose	196 mg/dL
Endotoxin	<2 pg/mL
Procalcitonin	0.207
Ferritin	1713 ng/mL
Soluble IL-2R	1235 U/mL
HbA1c	5.7%
IgG	1035 mg/dL
IgA	129 mg/dL
IgM	34 mg/dL
Anti-nuclear antibody	<40
HBs antigen	(—)
HCV antibody	(—)
HIV antibody	(—)
TPHA	(—)
*Urea *	
	>1.030
pH	6.5
Protein	(2+)
Sugar	(—)
Ketone	(—)
Occult blood	(1+)

Lym.: lymphocyte; WBC: white blood cells; PT: prothrombin time; FDP: fibrin/fibrinogen degradation products; BUN: blood urea nitrogen; ALP: alkaline phosphatase; *γ*-GTP: *γ*-glutamyltransferase; T-Bil: total bilirubin; CRP: C-reactive protein; IL-2R: interleukin-2 receptor; Ig: immunoglobulin; HBs: hepatitis B surface; HCV: hepatitis C virus; HIV: human immunodeficiency virus; TPHA: treponema pallidum latex agglutination.
